# Challenges in diagnosis and management of pulmonary valve endocarditis associated with autoimmune lymphoproliferative syndrome (ALPS): A case report and literature review

**DOI:** 10.1016/j.radcr.2025.09.067

**Published:** 2025-10-16

**Authors:** Youssef Lahmouz, Sara Ahchouch, Hajar Moukane, Mehdi Bamous, Najat Mouine, Aatif Benyass

**Affiliations:** Cardiology Center, Mohammed V Military Instruction Hospital of Rabat, Mohammed V University, Rabat, Morocco

**Keywords:** Endocarditis, Pulmonary regurgitation, Echocardiography, ALPS

## Abstract

Isolated infection of the pulmonary valve is rarely encountered. The diagnosis is not considered based on clinical presentation or even on transthoracic echocardiography. A conservative approach is generally recommended for most patients with infective endocarditis. Our patient also had an autoimmune lymphoproliferative syndrome (ALPS), described as a rare hereditary disorder of lymphocyte homeostasis, resulting from mutations in the Fas apoptosis pathway. The elevated level of double-negative T cells (DNT) is considered a major diagnostic marker for this rare pathology. An 18-year-old female was admitted to our institution for prolonged febrile illness persisting for 2 years. She also suffered from respiratory symptoms for 2 months treated with amoxicillin without clear amelioration. After a thorough assessment, the patient was diagnosed with pulmonary valve endocarditis associated with autoimmune lymphoproliferative syndrome. Appropriate management, started by empirical antibiotics then intravenous immunoglobulin and prednisolone. Our patient underwent after 6 months of the follow up, pulmonary valve replacement by bioprosthesis. Endocarditis of the right side of the heart mostly affects the tricuspid valve, especially in cases involving drug users. Isolated infection of the pulmonary valve is rarely encountered. Accurate and early diagnosis of pulmonary valve endocarditis is essential for implementing appropriate management strategies. The autoimmune lymphoproliferative syndrome (ALPS) is a rare hereditary disorder of lymphocyte homeostasis, resulting from mutations in the Fas apoptosis pathway. Lymphoproliferation is the most common manifestation in ALPS, illustrated by lymphadenopathy, splenomegaly, and/or hepatomegaly persisting for over 6 months. Right‐Sided Infective Endocarditis affecting only the pulmonary valve is very rare. It should be considered to investigate for right-sided infective endocarditis and echocardiography leads the diagnosis.

## Introduction

Infective endocarditis (IE) involving the pulmonary valve is exceptionally rare, representing <2% of all IE cases. It is usually associated with congenital heart disease, intravenous drug use, or indwelling catheters. Autoimmune lymphoproliferative syndrome (ALPS) is a rare disorder of lymphocyte apoptosis characterized by lymphadenopathy, splenomegaly, autoimmune cytopenias, and elevated double-negative T cells. The coexistence of pulmonary valve IE and ALPS has not been widely reported.

## Case report

An 18-year-old female was admitted for prolonged febrile illness persisting for 2 years, previously undiagnosed despite hospitalization in pediatrics. We noticed the use of cauterization as part of traditional local treatment after her discharge. Two months before admission, she developed respiratory symptoms treated unsuccessfully with amoxicillin. She denied intravenous drug use.

On admission, she was in poor general condition with low-grade fever (38°C) and pustular skin lesions on her arm. Hemodynamics were stable except for low blood pressure (100/55 mmHg), tachycardia (107 bpm), and oxygen saturation of 96% on room air. Cardiac auscultation revealed a harsh holosystolic murmur (grade 4/6) at the left sternal border and a diastolic murmur maximal at the pulmonary area. Abdominal examination revealed hepatosplenomegaly.

**Fig. 1:**
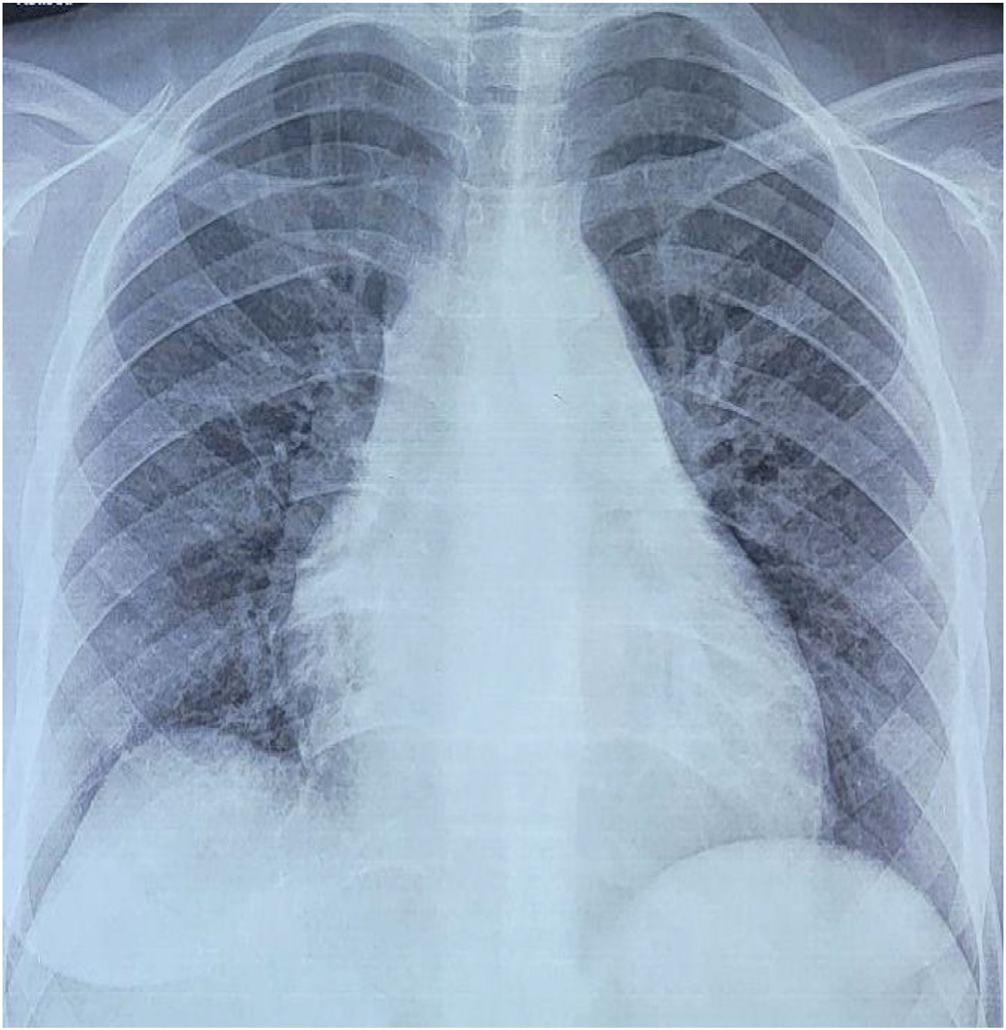
Frontal chest X-ray showed moderate cardiomegaly, leftward convexity of the mid-heart shadow.

**Fig. 2:**
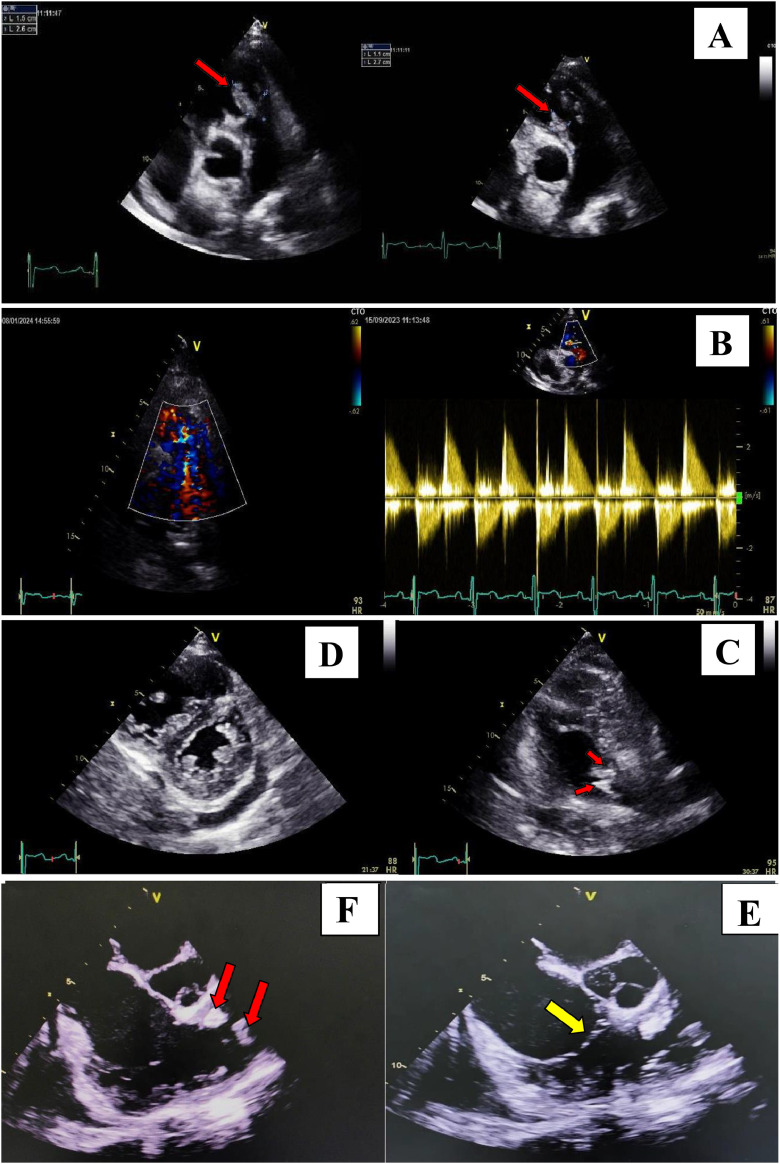
Transthoracic echocardiography (A, B, C, D) and transesophageal echocardiography (E, F) revealed 2 large vegetations (red arrows) attached to the pulmonary valve and severe pulmonary regurgitation. No vegetations were seen on the tricuspid valve (yellow arrow). The right ventricle was dilated with paradoxical septal motion with deteriorating right ventricle function (D).

ECG showed sinus rhythm with right bundle branch block. Chest X-ray demonstrated cardiomegaly (cardiothoracic index 0.55) with leftward mid-heart convexity (Fig. 1). Transthoracic echocardiography (TTE) revealed severe pulmonary regurgitation and 2 large vegetations (26 × 15 mm and 11 × 27 mm) attached to the pulmonary valve, associated with right ventricular dilatation and dysfunction (TAPSE 11 mm, S wave 7 cm/s) (Fig. 2 A-D). Transesophageal echocardiography confirmed these findings, with no involvement of the tricuspid or other valves (Fig. 2 E, F). Repeated blood cultures remained sterile.

Initial labs showed anemia (Hb 9.2 g/dL;12-16 g/dL), thrombocytopenia (109,000/mm³; 250000–400000/mm^3^), leukocytes (5300/mm³; 4.2–11.6 cells/mm^3^) and elevated CRP (42 mg/dL; <5 mg/dL). CT imaging confirmed hepatosplenomegaly, pulmonary emboli, and mediastinal adenopathy. A diagnosis of pulmonary valve IE was made, and she was started on ceftriaxone, amoxicillin, and gentamicin. She became afebrile within 3 days.

After 7 days, fever relapsed (39°C). Repeat TTE showed unchanged findings.

New labs revealed worsening pancytopenia (Hb 7.5 g/dL, platelets 9000/mm³, neutrophils 0/mm³). She was switched to imipenem, vancomycin, and fluconazole. Additional workup showed autoimmune hemolytic anemia (positive Coombs test, elevated LDH, undetectable haptoglobin) and autoimmune neutropenia. Bone marrow and vitamin B12 levels were normal. The combination of cytopenias, hepatosplenomegaly, and lymphadenopathy suggested ALPS. Flow cytometry confirmed the diagnosis with double-negative T cells at 4% (normal <2.5%).

**Fig. 3:**
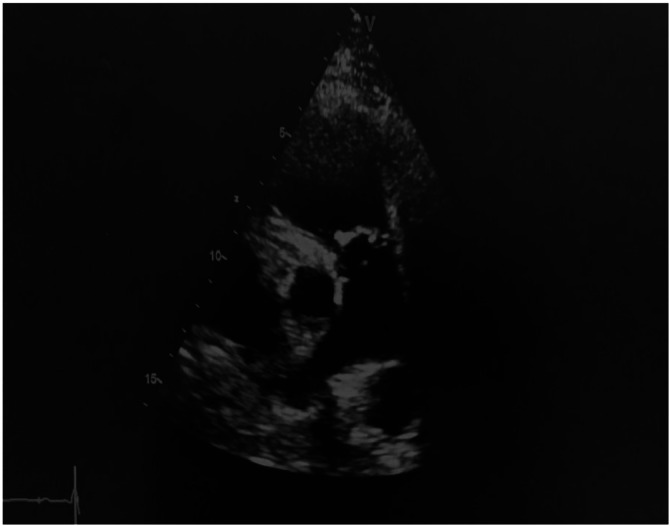
Transthoracic echocardiography at the 6-month revealed absence of the vegetations.

She responded to G-CSF, IV Immunoglobulins, corticosteroids, and completed 6 weeks of antibiotics. Follow-up TTE showed regression of vegetations but persistent severe pulmonary regurgitation Fig. 3. At 6 months, despite improvement in RV function (TAPSE 22 mm, S wave 13 cm/s), RV dilation persisted. Cardiac MRI confirmed these findings.

The Heart Team opted for pulmonary valve replacement with bioprosthesis. Surgery was uneventful, and she was discharged on postoperative day 6 in good condition.

## Discussion

We report a case of isolated pulmonary valve endocarditis, complicated by pulmonary septic embolism. The significance of this observation lies in the rarity of this unusual site of right‐Sided Infective Endocarditis and in the pediatric patient. The delay of the diagnosis in our patient caused chronic massive pulmonary regurgitation and leads to right ventricular (RV) volume overload, which increases RV preload. It also causes volume overload in the proximal central pulmonary artery (PA), contributing to vascular remodeling and eventually increasing RV afterload, affecting the right ventricle function [1].

Right-sided endocarditis accounts for approximately 5% to 10% of infective endocarditis cases [2]. Pulmonary valve involvement is most often associated with tricuspid involvement and exceptionally isolated, representing less than 2% of infective endocarditis cases overall. Most reported cases involve children with congenital malformations, drug users, patients with intravenous catheters, or even catheters in the pulmonary artery [2,3].

Clinical manifestations can be remarkable with pulmonary embolic events occurring in at least 40% of cases [4]. Bacteriologically, in 25% of cases, Coagulase-negative Staphylococci species were the most common micro-organisms identified [5].

Transthoracic echocardiography (TTE) has been reported to achieve a diagnostic rate of 91% in isolated pulmonary valve infective endocarditis cases [6]. Preliminary studies suggest that transesophageal echocardiography (TEE) may enhance the detection of vegetations.

Nevertheless, studying the pulmonary valve with TEE can be challenging, even for experienced echocardiographers, due to its anatomical position as the most anteriorly located valve and its distance from the TEE probe, which can limit optimal visualization [7,8].

However, TEE proved advantageous in visualizing other cardiac valves and assessing device lead involvement.

The role of surgery in isolated pulmonary valve endocarditis remains unclear. It is a significant challenge for cardiac surgeons due to poor postoperative adherence and a high rate of relapse. Surgical options include debridement of the infected area, excision of vegetation with either valve preservation or repair, or valve replacement [9].

The Autoimmune Lymphoproliferative Syndrome (ALPS) is a rare hereditary disorder of lymphocyte homeostasis, resulting from mutations in the Fas apoptosis pathway [10].

The elevated level of double-negative T cells (DNT) is considered a major diagnostic marker for this rare pathology [11]. Lymphoproliferation is the most common manifestation in pediatric population, illustrated by lymphadenopathy, splenomegaly, and/or hepatomegaly persisting for over 6 months [11]. Accordingly, patients with probable ALPS should be treated by glucocorticoids with cyclosporine or rituximab or high-dose intravenous immune globulin [12]. Morbidity and mortality in ALPS depend on the severity of the autoimmune disease and development of lymphoma [13].

## Conclusion

We report a rare case of pulmonary valve IE revealing ALPS in a young woman. Careful integration of cardiological, infectious, and immunological findings was crucial for diagnosis and management. Early recognition of underlying immune dysregulation is essential in atypical IE presentations, as it directly impacts treatment and prognosis.

## Patient consent

Written informed consent for the publication of this case report was obtained from the patient.
